# Severe Hepatic Insulin Resistance Induces Vascular Dysfunction: Improvement by Liver-Specific Insulin Receptor Isoform A Gene Therapy in a Murine Diabetic Model

**DOI:** 10.3390/cells10082035

**Published:** 2021-08-09

**Authors:** Almudena Gómez-Hernández, Natalia de las Heras, Andrea R. López-Pastor, Gema García-Gómez, Jorge Infante-Menéndez, Paula González-López, Tamara González-Illanes, Vicente Lahera, Manuel Benito, Óscar Escribano

**Affiliations:** 1 Laboratory of Hepatic and Cardiovascular Diseases, Biochemistry and Molecular Biology Department, School of Pharmacy, Complutense University of Madrid, 28040 Madrid, Spain; andreara@ucm.es (A.R.L.-P.); jorgeinf@ucm.es (J.I.-M.); pgonza23@ucm.es (P.G.-L.); tagonz02@ucm.es (T.G.-I.); 2 Department of Physiology, School of Medicine, Complutense University of Madrid, 28040 Madrid, Spain; nataliaheras@med.ucm.es (N.d.l.H.); vlahera@med.ucm.es (V.L.); 3 Laboratory of Diabetes and Obesity, Biochemistry and Molecular Biology Department, School of Pharmacy, Complutense University of Madrid, 28040 Madrid, Spain; ggarciag@ucm.es (G.G.-G.); mbenito@ucm.es (M.B.); 4 Centro de Investigación Biomédica en Red (CIBER) de Diabetes y Enfermedades Metabólicas Asociadas (CIBERDEM), 28040 Madrid, Spain; 5 Mechanisms of Insulin Resistance (MOIR2), General Direction of Universities and Investigation (CCMM), 28040 Madrid, Spain

**Keywords:** insulin resistance, endothelial dysfunction, atherosclerosis, gene therapy

## Abstract

Background: Cardiovascular dysfunction is linked to insulin-resistant states. In this paper, we analyzed whether the severe hepatic insulin resistance of an inducible liver-specific insulin receptor knockout (iLIRKO) might generate vascular insulin resistance and dysfunction, and whether insulin receptor (IR) isoforms gene therapy might revert it. Methods: We studied in vivo insulin signaling in aorta artery and heart from iLIRKO. Vascular reactivity and the mRNA levels of genes involved in vascular dysfunction were analyzed in thoracic aorta rings by qRT-PCR. Finally, iLIRKO mice were treated with hepatic-specific gene therapy to analyze vascular dysfunction improvement. Results: Our results suggest that severe hepatic insulin resistance was expanded to cardiovascular tissues. This vascular insulin resistance observed in aorta artery from iLIRKO mice correlated with a reduction in both PI3K/AKT/eNOS and p42/44 MAPK pathways, and it might be implicated in their vascular alterations characterized by endothelial dysfunction, hypercontractility and eNOS/iNOS levels’ imbalance. Finally, regarding long-term hepatic expression of IR isoforms, IRA was more efficient than IRB in the improvement of vascular dysfunction observed in iLIRKO mice. Conclusion: Severe hepatic insulin resistance is sufficient to produce cardiovascular insulin resistance and dysfunction. Long-term hepatic expression of IRA restored the vascular damage observed in iLIRKO mice.

## 1. Introduction

Cardiovascular diseases (CVDs) are among the major causes of morbidity and mortality in Western countries, and they are closely related to obesity, which is currently considered a global pandemic. Indeed, suffering obesity increases the risk of CVDs 2- to 3-fold [1]. Obesity is a chronic and multifactorial disease characterized by an increased body weight with excessive fat accumulation, as well as low grade inflammation [2,3]. This condition is also associated with insulin resistance, type 2 diabetes mellitus (T2DM) and dyslipidemia, characterized by increased serum triglycerides, decreased high-density lipoproteins (HDL) cholesterol and increased small dense low-density lipoprotein (LDL) particles [4]. Despite the alarming prevalence of obesity, defining the pathogenic links between insulin resistance and the associated cardiovascular risk is currently poorly understood [5,6].

Insulin resistance affects several tissues with relevant roles in glucose homeostasis such as the liver, skeletal muscle, and the adipose organs. Certainly, hepatic insulin resistance is manifested by the blunted ability to regulate the suppression of hepatic glucose production; therefore, it is accompanied by clear hyperglycemia [7]. The insulin receptor (IR) belongs to subclass II of the tyrosine kinase receptor superfamily and is essential in glucose homeostasis regulation [8]. In mammals, alternative splicing of exon 11 generates IRA and IRB isoforms lacking or containing 12 additional amino acids, respectively, located immediately downstream of the ligand binding domain [9]. This sequence does not affect insulin binding affinity [9,10], but IRA has approximately 10-fold higher affinity for IGF-I and IGF-II than IRB [11], and it is predominantly expressed during fetal development, where it mediates IGF-II effects [12]. In addition, it has also been described that an increased IRA/IRB ratio can be observed in beta cells under certain pathophysiological conditions, such as insulin resistance [13]. Conversely, IRB is predominantly expressed in adult tissues, including the liver, where it mediates the metabolic effects of insulin [11]. Although insulin signaling is not required for hepatic glucose uptake, in vitro studies in neonatal hepatocytes and pancreatic beta cells demonstrate that IRA plays a direct role favoring glucose uptake, since it is constitutively associated with endogenous glucose transporters (GLUT1 and GLUT2) [13,14]. Therefore, differences in the capability of glucose uptake can be associated with the presence or absence of IR isoforms, or with changes in the ratio between them.

In recent studies, to elucidate the role of IR isoforms in glucose and lipid metabolism, we took advantage of the capacity of adeno-associated vectors serotype 8 (AAV8) to specifically transfer recombinant genes to the liver [15]. In our hands, the hepatic expression of IRA through gene therapy approaches with AAV8 resulted in an improvement in glucose tolerance, even with a regression in beta cell hyperplasia [16], as well as in hepatic steatosis [17].

With this background, in this paper, we analyzed whether the severe hepatic insulin resistance of inducible liver-specific insulin receptor knockout (iLIRKO) mice expands to the cardiovascular system, inducing insulin resistance in aorta artery and heart. Moreover, we studied whether iLIRKO mice developed vascular damage and defects in vascular reactivity and, finally, we designed a hepatic-specific gene therapy approach to differentially express both IR isoforms. Our results demonstrate that hepatic IRA expression improves the vascular damage observed in iLIRKO mice.

## 2. Materials and Methods

### 2.1. Mice

*Insr*^lox/lox^ (hereafter IR^(lox/lox)^) mice were created by homologous recombination using an insulin receptor gene-targeting vector with loxP sites flanking exon 4 [18]. For liver-specific deletion, we used transgenic mice with the tamoxifen-dependent Cre-ER^T2^ recombinase coding sequence under control of the albumin promoter. The iLIRKO mice were generated by crossing C57Bl/6 IR^(lox/lox)^ and heterozygous C57Bl/6 Alb-Cre-ER^T2^; littermate IR^(lox/lox)^ were used as control. Genotyping of the IR^(lox/lox)^ and Alb-Cre-ER^T2^ transgenic mice was performed by PCR using genomic DNA isolated from the tip of the tail of 3- to 4-week-old mice, as previously described [13]. After weaning, iLIRKO and control mice (IR^(lox/lox)^) were fed with a soy-free diet (RMS-0909-US-EN-02-DS-2016; Harlan Teklad, Barcelona, Spain) for two weeks, followed by two weeks of tamoxifen diet (TD.09327, Harlan Teklad, Barcelona, Spain) in order to induce translocation of Cre to the nucleus. Following this, animals were fed with a standard chow ad libitum (Figure S1A). Only male animals were studied and maintained on a 12 h light-dark cycle. All animal experimentation was conducted in accordance with the accepted standards of animal use approved by the Complutense University of Madrid Ethics Committee (approval code: PROEX 188/16).

In vivo insulin signaling and vascular reactivity studies were performed in four groups: WT and iLIRKO at 6 months of age (*n* = 11–14 and *n* = 17–19, respectively) and WT and iLIRKO at 12 months (*n* = 18–21 and *n* = 9–12, respectively).

A second study was included in this paper using iLIRKO mice treated with recombinant AAV vectors with IRA o IRB isoforms (Figure S1B). In addition, to reestablish hepatic insulin signaling and glucose metabolism alterations, the aim was to analyze whether the presence of these isoforms might improve associated vascular damage.

Regarding the latter study, we previously performed viral constructors and vector production and purification. Recombinant AAV vectors were constructed with a transgene cassette coding sequence for the individual-spliced single-chain isoforms of *INSR* either containing or lacking exon 11 (IRB and IRA, respectively), or the reporter *luc* under the regulation of a liver-specific promoter, α1-antitrypsin (AAT). Coding sequences for the human IR isoforms were a generous gift of Dr. C.R. Kahn (Joslin Diabetes Center, Boston, MA, USA). The transgene cassette was flanked by AAV2 wild-type inverted terminal repeats. rAAV8 vectors were produced as previously described [19].

AAV administration was injected intravenously in 5-month-old mice. For all procedures, animals were anaesthetized by intraperitoneal (i.p.) injection of a mixture of xylacine (Rompun 2%, Bayer, Leverkusen, Germany) and ketamin (Imalgene 50, Merial, Lyon, France) at 1:9 *v*/*v*.

### 2.2. In Vivo Insulin Signaling Studies

For in vivo insulin signaling studies, fasted mice were injected with 1 U/kg body weight of human insulin (Novo Nordisk, Madrid, Spain) into the peritoneal cavity. After 10 min, tissues were removed and immediately frozen in liquid nitrogen. By Western blot, we studied insulin receptor β subunit (IRβ), phospho-AKT (Ser473), phospho-p44/42 MAPK (Ser202/Tyr204) and phospho-p70S6K (Thr389) in aorta artery and heart.

### 2.3. Western Blot

Western blot analysis was performed on tissue homogenates as described [13]. The antibodies used were anti-phospho-AKT (Ser473), anti-phospho-p44/p42-MAPK (Thr202/Tyr204) and anti-phospho-p70S6K (Thr389) from Cell Signaling Technology (Danvers, MA, USA); anti-IRβ from Santa Cruz Biotechnology (Dallas, TX, USA) and anti-β-actin from Sigma-Aldrich Corp (St. Louis, MO, USA). Fifty micrograms of whole proteins were separated on an 8–15% SDS-PAGE and transferred to a polyvinylidene fluoride membrane (Immobilon P; Millipore, Burlington, MA, USA). The membrane was blocked in washing solution with 5% non-fat dried milk for 60 min at 37 °C and then incubated with 1 μg/mL primary antibody overnight at 4 °C. The primary antibodies were immunodetected using a horseradish peroxidase-conjugated secondary antibody for 60 min at 37 °C. The bands were detected with a chemiluminescent system (ECL; GE Healthcare, Piscataway, NJ, USA) and exposed to X-ray film. The band intensities were quantified using ImageJ v1.52k software (http://rsb.info.nih.gov/ij, accessed on 20 January 2021).

### 2.4. Vascular Reactivity

Vascular function was studied in aortic rings from control and iLIRKO mice at 6 and 12 months of age. Endothelial function was studied by evaluating endothelium-dependent relaxations to acetylcholine (ACh; 10^−9^ to 10^−5^ mol/L), endothelium-independent relaxations induced by sodium nitroprusside (SNP; 10^−10^ to 10^−7^ mol/L) and relaxation to insulin (10^−10^ to 10^−6^ mol/L) in phenylephrine (PE; 10^−6^ mol/L) precontracted rings. Moreover, we analyzed the contractile response to angiotensin II (Ang II; 10^−6^ mol/L), angiotensin I (Ang I; 10^−6^ mol/L) and U46619, a thromboxane A2 (TXA2) analogue (U46619; 10^−6^ to 10^−9^ mol/L).

### 2.5. Histological Analysis

Aortic roots were optimal-cutting-temperature embedded, and sections of 7 μm were Oil Red O/hematoxylin stained to measure lipid depot (Image-Pro Plus software, Media Cybernetics, Inc. Rockville, MD, USA) and % lesion area/total area (Image J v1.52k software, http://rsb.info.nih.gov/ij, accessed on 20 January 2021).

### 2.6. Extraction of mRNA and qRT-PCR

Total RNA was extracted from several tissues by the TRIzol method (Invitrogen, Carlsbad, CA, USA) and quantified by absorbance at 260 nm in duplicate. One microgram of RNA was used to perform the reverse transcription with a high-capacity cDNA archive kit (Applied Biosystems, Foster City, CA, USA). Gene expression was analyzed by real-time quantitative PCR (qRT-PCR) as described [13]. Thus, the relative abundance of mRNA targets, normalized to endogenous gene and relative to the control, is given by real-time quantitative (RQ) = 2^−^^ΔΔCt^ [ΔCt (cycle threshold) = Ct (target gene) − Ct (*Gapdh*); ΔΔCt = ΔCt for any sample − ΔCt for the control]. Amplification of *Gapdh* was used in the same reaction of all samples as an internal control.

### 2.7. Statistical Analysis

Data are presented as mean ± standard error of the mean (SEM). Normality of these variables was tested with a Shapiro–Wilk test. Differences were assessed using unpaired two-tailed Student’s *t*-tests and unpaired non-parametric Mann–Whitney U tests, as appropriate. The null hypothesis was rejected when the *p* value was <0.05. The software used for the analyses was GraphPad Prism v8.0 software (GraphPad Software Inc., San Diego, CA, USA).

## 3. Results

### 3.1. Hepatic Insulin Resistance Extends to Cardiovascular System

**Figure 1:**
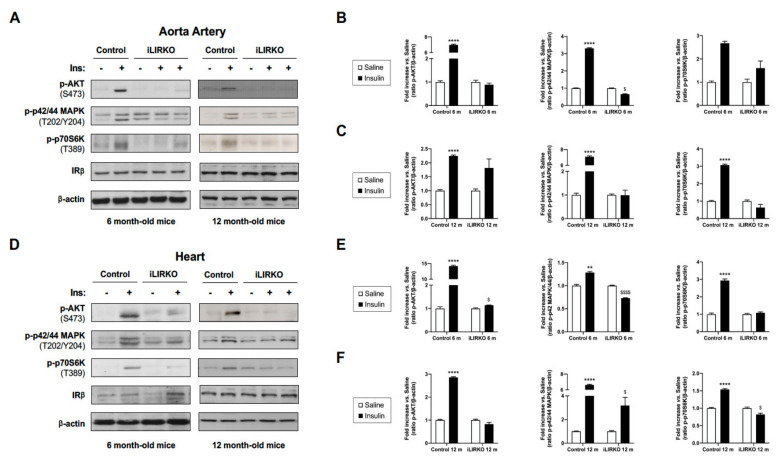
Hepatic insulin resistance expands to the cardiovascular system: representative gels (**A**,**D**) and quantification of in vivo insulin signaling studies (**B**,**C**,**E**,**F**) performed in control and iLIRKO mice at 6 and 12 months of age. After 10 min of human insulin injection at 1 U/kg body weight, aorta artery and heart were removed. Protein extracts from aorta artery and heart were analyzed by Western blot with anti-IRβ, anti-phospho-AKT (Ser473), anti-phospho-p42/44 MAPK (Thr202/Tyr204), anti-phospho-p70S6K (Thr389) and anti-β-actin antibodies. A representative experiment of three is shown. Results are expressed as mean ± SEM. *n* = 3–6 per group. The statistical significance was represented as ** (*p* < 0.01) and **** (*p* < 0.0001) vs. saline control mice; ^$^ (*p* < 0.05), and ^$$$$^ (*p* < 0.0001) vs. saline iLIRKO mice.

We used iLIRKO mice, a model of severe hepatic insulin resistance and T2DM, as previously described [13,16]. iLIRKO presented impaired glucose tolerance, fasted hyperinsulinemia, together with compensatory pancreatic hyperplasia, without showing any hepatic dysfunction. In this paper, we wanted to demonstrate whether hepatic insulin resistance might be expanded to cardiovascular tissues. For this objective, we analyzed in vivo insulin signaling in aorta artery and heart from control and iLIRKO mice at 6 and 12 months of age (Figure 1). By Western blot analysis, we first confirmed that, in aorta artery and heart, IRβ protein levels were remarkably similar between control and iLIRKO mice at 6 and 12 months of age (Figure 1A). In addition, we observed a significant decrease in insulin-induced AKT, p42/44 MAPK and p70S6K phosphorylation in aorta artery and heart from iLIRKO mice compared to control mice at 6 and 12 months of age, demonstrating that hepatic insulin resistance impairs cardiovascular insulin signaling (Figure 1B,C,E,F).

### 3.2. Hepatic Insulin Resistance Provokes Vascular Dysfunction

**Figure 2:**
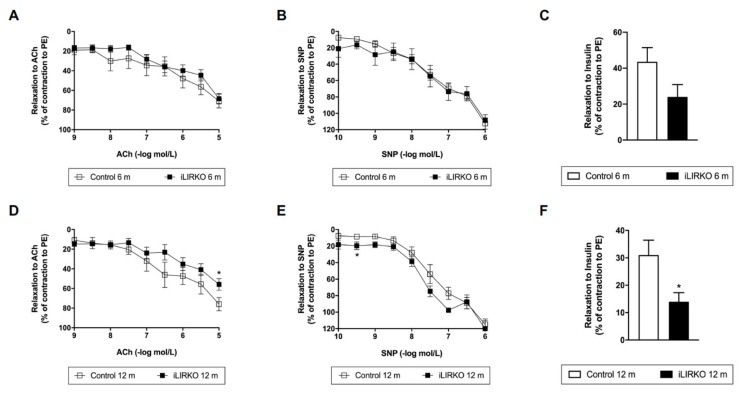
Vascular relaxation analysis in control and iLIRKO mice: relaxations to acetylcholine (ACh) (10^−^^9^ to 10^−5^ mol/L) (**A**,**D**), sodium nitroprusside (SNP) (10^−^^10^ to 10^−6^ mol/L) (**B**,**E**) or insulin (10^−6^ mol/L) (**C**,**F**) in phenylephrine (PE) (10^−^^6^ mol/L) precontracted rings, respectively. (**A**) Control (*n* = 8) and iLIRKO (*n* = 14) mice. (**B**) Control (*n* = 13–15) and iLIRKO (*n* = 6) mice. (**C**) Control (*n* = 13) and iLIRKO (*n* = 6) mice. (**D**) Control (*n* = 7–10) and iLIRKO (*n* = 7–8) mice. (**E**) Control (*n* = 9) and iLIRKO (*n* = 2–3) mice. (**F**) Control (*n* = 7) and iLIRKO (*n* = 5) mice. Results are expressed as mean ± SEM. The statistical significance was represented as * (*p* < 0.05) vs. control mice.

Thus, we analyzed vascular dysfunction in aortic rings from iLIRKO mice at 6 and 12 months of age as compared with their controls (Figure 2A,D). First, we studied endothelial function as endothelium-dependent relaxation to ACh. In this regard, we observed that a relaxing response to ACh was significantly lower in aortic rings previously precontracted with PE from 12-month-old iLIRKO mice vs. control (Figure 2D). These results might suggest that 12-month-old iLIRKO mice showed endothelial dysfunction. In contrast, endothelium-independent relaxation to SNP was comparable in the four studied groups (~100%) (Figure 2B,E).

On the other hand, we also analyzed insulin-induced relaxation in PE precontracted rings from control and iLIRKO mice (Figure 2C,F). A significantly lower relaxing response to insulin was observed in aortic rings from 12-month-old iLIRKO compared to control mice (Figure 2F). These results are consistent with those previously described in Figure 1.

**Figure 3:**
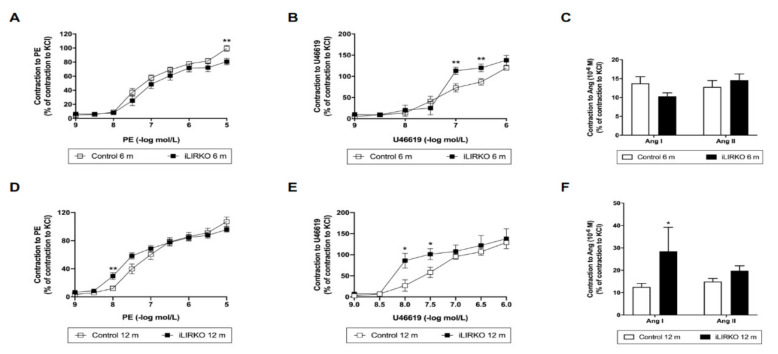
Vascular contraction analysis in control and iLIRKO mice: contractile responses to PE (**A**,**D**), U46619 (**B**,**E**) and Ang I/II (**C**,**D**). (**A**) Control (*n* = 21) and iLIRKO (*n* = 12) mice. (**B**) Control (*n* = 11) and iLIRKO (*n* = 5) mice. (**C**) Control (*n* = 14–16) and iLIRKO (*n* = 5) mice. (**D**) Control (*n* = 14) and iLIRKO (*n* = 11) mice. (**E**) Control (*n* = 4–6) and iLIRKO (*n* = 4) mice. (**F**) Control (*n* = 9–10) and iLIRKO (*n* = 3) mice. Results are expressed as mean ± SEM. The statistical significance was represented as * (*p* < 0.05) and ** (*p* < 0.01) vs. control mice.

We also evaluated the vasoconstrictor response to PE, U46619, Ang I and Ang II in aortic rings from the four studied groups. Constrictor responses to PE, Ang I and U46619 in aortic rings were higher in 12-month-old iLIRKO mice compared with each control group (Figure 3). As seen above regarding endothelial dysfunction, 12-month-old iLIRKO mice showed vascular dysfunction.

### 3.3. Long-Term Hepatic Expression of IRA Improves Vascular Dysfunction in iLIRKO Mice

**Figure 4:**
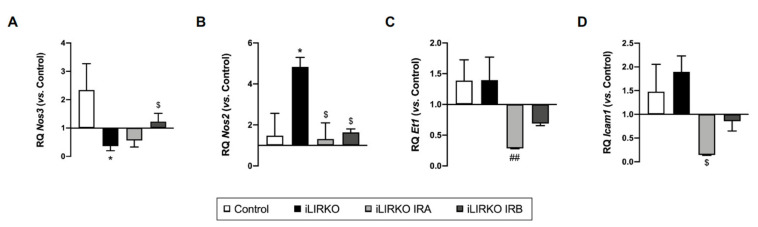
Long-term hepatic expression of IR isoforms alters mRNA levels of genes involved in vascular dysfunction: *Nos3* (**A**), *Nos2* (**B**), *Et1* (**C**) and Icam1 (**D**) mRNA levels by qRT-PCR on aorta artery from control (*n* = 2–6), iLIRKO (*n* = 3–11), iLIRKO IRA (*n* = 3), iLIRKO IRB (*n* = 3). Results are expressed as mean ± SEM. The statistical significance was represented as * (*p* < 0.05) vs. control mice; ^$^ (*p* < 0.05) vs. iLIRKO mice; ^##^ (*p* < 0.01) vs. iLIRKO IRB mice.

As mentioned above, iLIRKO mice develop cardiovascular insulin resistance and endothelial dysfunction. In a previous study carried out in iLIRKO mice, we reported that in vivo long-term AAV-mediated hepatic expression of IRA could act as a glucose uptake promoter, regulating hyperglycaemia, improving glucose homeostasis, precluding beta cell mass expansion and, therefore, avoiding the final beta cell failure [16]. In this sense, we also wanted to analyze whether long-term hepatic expression of IRA could improve the vascular damage observed in iLIRKO mice. To carry out this objective, we analyzed mRNA expression levels of genes involved in the vascular dysfunction by qRT-PCR. AAV-IRA-injected iLIRKO (iLIRKO IRA) mice showed a significant reduction in inducible nitric oxide synthase (*Nos2*) and intercellular adhesion molecule 1 (*Icam1*) mRNA levels compared to iLIRKO mice, and a significant decrease in endothelin 1 (*Et1*) mRNA levels vs. AAV-IRB-injected iLIRKO (iLIRKO IRB) mice (Figure 4B–D). On the other hand, iLIRKO IRB also showed a significant decrease in *Nos2* expression, in addition to a significant increase in endothelial nitric oxide synthase (*Nos3*) mRNA expression levels compared to iLIRKO mice (Figure 4A,B).

**Figure 5:**
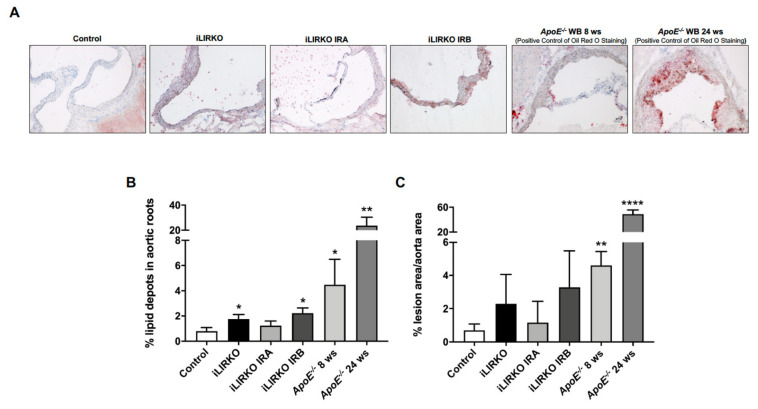
Long-term hepatic expression of IRA improves lipid depot in aortic roots: (**A**) representative microphotographs of Oil Red O/hematoxylin staining in aortic root sections from control (*n* = 3), iLIRKO (*n* = 3), iLIRKO IRA (*n* = 3), iLIRKO IRB (*n* = 3). As a reference, we used sections of aortic root from *ApoE^−/−^* mice under 8 weeks (*n* = 3) and 24 weeks (*n* = 5) of Western diet. Quantifications of lipid depot (**B**) and lesion area (**C**) in aortic roots from the six groups studied. Results are expressed as mean ± SEM. The statistical significance was represented as * (*p* < 0.05), ** (*p* < 0.01), **** (*p* < 0.0001) vs. control mice.

Moreover, we also analyzed lipid accumulation and injury in aortic roots. Bearing in mind that the *ApoE^−/−^* mouse model has been widely used in atherosclerosis research, we utilized these mice as positive controls. Our findings showed that iLIRKO IRA had a lower lipid depot and lesion area in aortic roots compared to the iLIRKO group, whereas both parameters were remarkably similar between iLIRKO and iLIRKO IRB mice (Figure 5).

## 4. Discussion

Endothelial dysfunction is linked to insulin-resistant states, including T2DM, obesity and metabolic syndrome [20]. Insulin resistance increases the susceptibility of patients to cardiovascular complications, such as atherosclerosis, stroke, coronary heart disease and hypertension.

With the idea of being able to delve into the mechanisms involved in insulin resistance, different experimental models have been developed in tissue-specific insulin receptor-deficient mice using Cre-loxP technology [18,21,22,23,24,25]. One of them has been the deficient model of the insulin receptor in the liver specifically (LIRKO) [25], which is characterized by alterations in glucose metabolism, together with compensatory hyperinsulinemia. Furthermore, a later study showed, in these same LIRKO mice, that hepatic insulin resistance was sufficient to produce dyslipidemia and susceptibility to atherosclerosis [26]. In this model, insulin resistance caused a proatherogenic distribution of serum cholesterol, with a significant decrease in HDL cholesterol and an increase in non-HDL cholesterol. Diverse analogies were found, with dyslipidemia associated with metabolic syndrome in humans, including increased secretion of ApoB by decreased intracellular degradation of ApoB [27], alteration in the expression of genes involved in the lipogenic response to insulin [28] and a catabolism defect of ApoB-containing lipoproteins [29]. Insulin resistance, in addition to increasing VLDL and reducing HDL levels, can modify the composition of LDL. Therefore, small, dense LDL may be more atherogenic than an equal number of larger cholesteryl ester-enriched LDL, since small dense LDL may be more liable to oxidation or may more readily penetrate and stick to the ECM of the artery wall [30,31]. All these data highlight the central role of insulin resistance in the development of dyslipidemia and atherosclerosis in patients with metabolic syndrome.

However, the diabetic phenotype observed in LIRKO mice reverted with aging, suggesting some form of compensatory mechanism, possibly linked to the early onset of insulin resistance. In addition, these mice developed liver damage, with hyperplastic nodules that might have altered glucose consumption by the hepatocytes, probably leading to the regression of the diabetic phenotype [25]. All these results limited the understanding of the full impact of hepatic insulin resistance in the pathogenesis of T2DM. To better address the role of adult hepatic insulin resistance in the pathogenesis of T2DM, our group developed an inducible model in order to generate liver IR deficiency (iLIRKO) in young mice. iLIRKO mice presented progressive hepatic and extrahepatic insulin resistance without liver dysfunction. iLIRKO mice displayed hyperinsulinemia and increased beta cell mass, suggesting a liver–pancreas endocrine axis in which IGF-1 functions as a liver derived growth factor to promote compensatory pancreatic islet hyperplasia through IRA [13]. In order to explore the relationship between insulin resistance and cardiovascular dysfunction in our model, we analyzed these processes in aorta artery and heart. Our results demonstrate that severe hepatic insulin resistance is expanded to cardiovascular tissues and, in consequence, contributes to endothelial dysfunction.

Different manifestations of insulin resistance, including dyslipidemia, hyperglycemia, inflammation and obesity, may be intermediary mediators together to insulin resistance to generate endothelial dysfunction. Therefore, hyperlipidemia, hyperglycaemia and proinflammatory cytokines are known to selectively impair the PI3K/AKT/eNOS pathway, increase oxidative stress and enhance the release of ET-1 by intact or heightened MAPK-ET-1 pathway from the endothelium [32]. Insulin-resistant states are associated with metabolic abnormalities that include glucotoxicity, lipotoxicity and inflammation, and which also lead to endothelial dysfunction. The latter term means a maladapted endothelial phenotype characterized by less nitric oxide (NO) bioavailability, abnormal vasoreactivity, increased oxidative stress, elevated expression of endothelial activation markers and proinflammatory factors [33]. In this sense, 12-month-old-iLIRKO showed endothelial dysfunction and vascular insulin resistance with a reduction in PI3K/AKT pathway signaling. The endothelial dysfunction observed in iLIRKO mice was characterized by abnormal vasoreactivity, together with an elevated expression of markers of endothelial activation and reduced NO bioavailability.

There are different mouse models where the proximal nodes in insulin signaling (IR and IRS-1) are affected, thereby impairing both the PI3K and MAPK pathways. In the aorta artery from our model, we also observed a significant decrease in insulin signaling in the PI3K/AKT/eNOS and p42/44 MAPK pathways, together with a significant reduction in iNOS, without affecting ET-1 levels. Previous published results also described that both pathways might be affected. Therefore, the endothelial cell IR knockout mouse (VENIRKO) showed normal glucose homeostasis and normal vascular development, but altered ET-1 and eNOS expression, vascular insulin resistance and a modest alteration in the regulation of blood pressure and insulin sensitivity under a low-salt diet [34]. IRS-1- and IRS-2-deficient mice not only exhibited insulin resistance but also diminished eNOS activity [35]. These findings noted that complex interactions between insulin action, eNOS, ET-1 and oxidative stress affect the metabolic and vascular phenotype in these mice. In contrast, endothelial dysfunction was pronounced in other mouse models that exhibited selective impairment in insulin resistance. Genetic ablation of *Akt1* in high-fat-fed *ApoE^−/−^* mice leads to increased atherogenesis and endothelial dysfunction [36]. Obese Zucker rats demonstrate selective insulin resistance in PI3K-dependent signaling (with intact MAPK signaling) in the vasculature and, as a consequence, show an impaired NO-mediated vasodilation and augmented ET-1-mediated vasoconstriction in response to insulin [37]. Regarding the importance of the insulin pathway in abnormalities associated with insulin resistant states, it has been described that the restoring of AKT activity in LIRKO mice under an atherogenic diet normalized serum glucose and the distribution of serum cholesterol by increasing HDL and decreasing non-HDL cholesterol [6]. Therefore, in the metabolic syndrome, insulin resistance drives changes in cholesterol and glucose homeostasis, whereas hyperinsulinemia generate hypertriglyceridemia, producing the full complement of lipid abnormalities associated with metabolic syndrome in humans.

In this way, insulin-resistant subjects carrying IRS-1 polymorphisms showed that impaired endothelial insulin signaling and reduced NO activity contributed to endothelial dysfunction [38]. Moreover, in endothelial cells from subjects carrying the G972R-IRS-1 variant, insulin-mediated PI3-K/AKT/eNOS activation was reduced [39]. Similarly, activation of AKT or eNOS were decreased in vascular tissue from patients with diabetes when compared with non-diabetics [40].

Proper endothelial function implies the bioavailability of NO and a balance in the activity of eNOS and iNOS in vascular tissue [41]. Physiological levels of NO produced by eNOS represent a key element for vascular endothelial homeostasis, with it being a protective factor by its vasodilatory, antiaggregant and antiproliferative effects [42]. On the contrary, NO overproduction, due to the activation of iNOS under different stress conditions, leads to endothelial dysfunction and, in the late stages, to the development of atherosclerosis [43]. In the atherosclerotic plaque, macrophages express iNOS, producing large amounts of NO. It might generate peroxynitrites, favoring vasospasm and thrombogenesis. According to this, in aorta artery from iLIRKO, we also observed an imbalance in the levels of both NOS isoforms, with a significant decrease in eNOS and marked increase of iNOS. Furthermore, the key role of both NOS isoforms and their implication in endothelial dysfunction and atherosclerosis has not only been described in experimental models [44,45] but also in human atherosclerosis [46,47]. For instance, iNOS expression was induced in human coronary atherosclerotic plaque and correlated with different factors of instability, including complaints of angina at rest or the presence of thrombus after morphological examination [47].

In addition to endothelial dysfunction observed in aorta artery from 12-month-old- iLIRKO, a hypercontractility induced by PE and Ang I was noted. In this sense, it was reported that intracellular Ca^2+^ in response to PE via the voltage-dependent Ca^2+^ channel was increased in the aorta of obese Zucker rats [48,49]. Therefore, it is possible that hypercontractility in response to Ang II might be due not only to an increased Ca^2+^ sensitization of contraction but also to an enhanced intracellular Ca^2+^ mobilization. One of the mechanisms implicated might be Rho activation, inducing the proliferation of vascular smooth muscle cells (VSMCs) [50,51] and the suppression of eNOS expression in vascular endothelial cells [52]. Furthermore, Rho activates Rho kinase, which in turn phosphorylates and inactivates myosin light chain phosphatase (MLCP). As MLCP dephosphorylates myosin light chain and induces relaxation of VSMCs, inactivation of MLCP results in vasoconstriction. Thus, activation of Rho increases the sensitivity of VSMCs contraction to a given intracellular Ca^2+^ concentration [53].

Endothelial cells can also increase vascular tone by releasing endothelium-derived contracting factor (EDCF). The overproduction of EDCF contributes to the endothelial dysfunction that accompanies various vascular diseases. The increase in intracellular Ca^2+^ concentration initiates the release of EDCF. Downstream processes include activation of phospholipase A2, cyclooxygenases and the production of ROS and vasoconstrictor prostanoids (prostacyclin, TXA2 and other prostaglandins), which subsequently activate thromboxane–prostanoid (TP) receptors on the VSMCs, leading to contraction. EDCF-mediated responses are amplified in diabetic animals [54] and in human atherosclerosis [55]. Similarly, an increased contractile response by TXA2 analogue in aorta artery from iLIRKO mice was noted.

Other molecules, such as PAI-1, might be key in linking insulin resistance, dyslipidemia and cardiovascular complications. This molecule has been described as a marker for risk of premature CAD [56]. Both hepatic and endothelial cells produced more PAI-1 in response to high insulin levels. In both in vitro and in vivo, high levels of VLDL also stimulated an increase in PAI-1 synthesis and secretion [57,58]. Therefore, hyperinsulinemia inhibits fibrinolysis in individuals with insulin resistance, and intravenous insulin infusion stimulated PAI-1 secretion in humans [59].

On the other hand, it has been described that the dysregulation of some microRNA expression is involved in hepatic disease and its vascular complications. These miRNAs are secreted by hepatocytes to the bloodstream in extracellular vesicles in order to regulate gene expression in different tissues [60]. In this sense, miR-122 is the main miRNA in the liver and its dysregulation is clear in hepatic steatosis [61], and thus in insulin resistance, inducing, at least in part, lipid metabolism alterations and other vascular complications. Therefore, miR-122 levels are elevated in patients with hyperlipidemia and are associated with CAD [62] and acute coronary syndrome [63]. Other miRNAs such as miR-34a [64], miR-132 and miR-143 [65] are also altered in hepatic and vascular diseases.

The modifications in lifestyle, including calorie restriction and physical interventions, may involve enhanced insulin signaling, increased eNOS activity, decreased iNOS activity and reduced oxidative stress and inflammatory markers that lead to a balance between the vasoconstrictor and vasodilator actions of insulin [66,67,68].

In addition, pharmacological treatments for T2DM decrease insulin resistance and, therefore, significantly reduce cardiovascular events [69,70,71]. Metformin administration improved endothelium-dependent vasodilation and simultaneously diminished ET-1 circulating levels in insulin-resistant subjects [69]. Similarly, rosiglitazone, an insulin sensitizer, ameliorated macrovascular reactivity and ACh-mediated vasodilation in patients with metabolic syndrome [70].

Finally, in our iLIRKO model, we previously demonstrated that long-term hepatic expression of IRA could be a promising therapeutic approach for the treatment of T2DM, since this isoform was more efficient than IRB in improving glucose intolerance [16]. As occurs with antidiabetic drugs, in the current paper, we demonstrate that a liver-specific gene therapy approach with IRA not only ameliorates the diabetic phenotype but also improves vascular damage observed in iLIRKO mice.

## 5. Conclusions

Our results suggest that severe hepatic insulin resistance expanded to cardiovascular tissues, such as the heart and artery aorta. The vascular insulin resistance observed in aorta artery from iLIRKO mice was due to a reduction in both PI3K/AKT/eNOS and p42/44 MAPK signaling pathways. This impairment might be implicated in the vascular alterations characterized by endothelial dysfunction hypercontractility and imbalanced eNOS/iNOS levels. Finally, long-term hepatic expression of IRA not only restored glucose metabolism abnormalities and diabetic phenotype but also improved the vascular damage observed in iLIRKO mice.

## Data Availability

Not applicable.

## Supplementary Materials

The following are available online at https://www.mdpi.com/article/10.3390/cells10082035/s1, Figure S1: Graphical schemes of iLIRKO model and the gene therapy protocol.
