# Supplementary material for: Severe Hepatic Insulin Resistance Induces Vascular Dysfunction: Improvement by Liver-Specific Insulin Receptor Isoform A Gene Therapy in a Murine Diabetic Model
Source: Cells. 2021 Aug 9;10(8):2035. doi: 10.3390/cells10082035 (PMC8392327; doi:10.3390/cells10082035)
Supplement: Supplementary file 1 [file cells-10-02035-s001.zip › cells-1324503-supplementary.pdf]

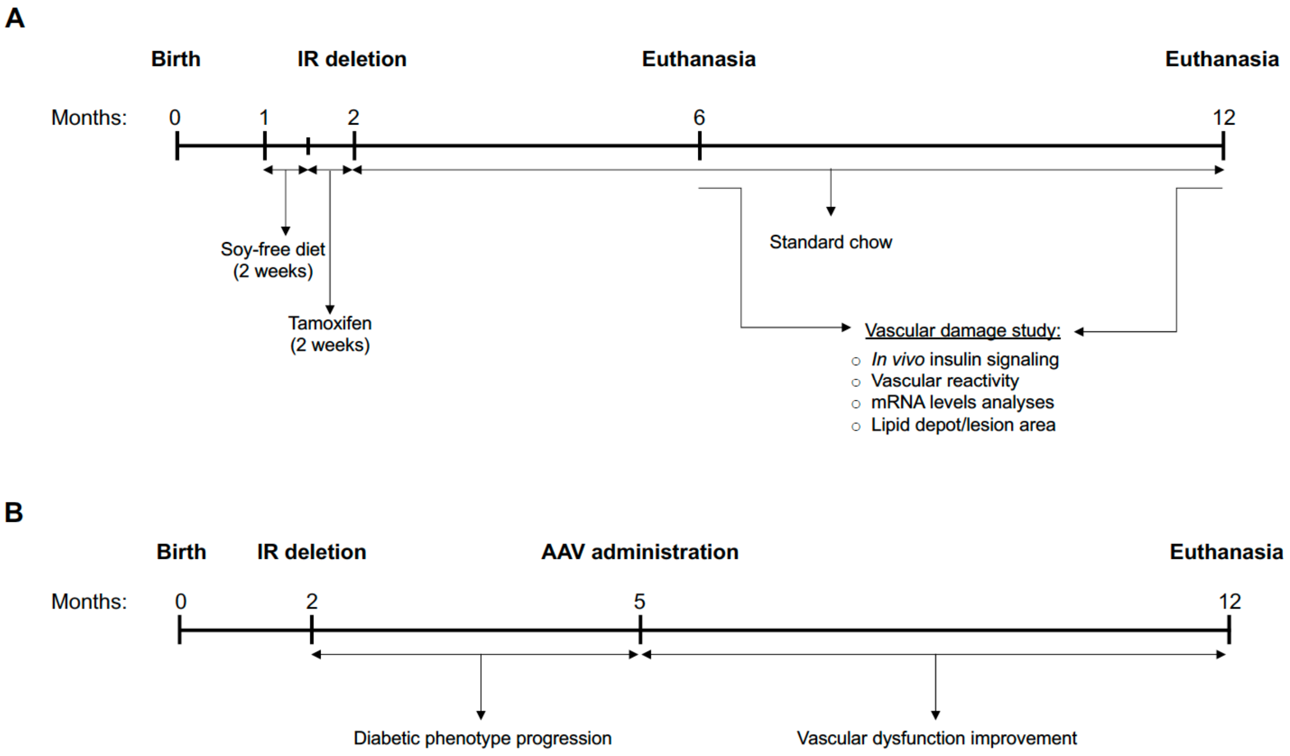

Figure S1. Graphical schemes of iLIRKO model and the gene therapy protocol. (**A**) Timeline of diets and Tamoxifen administration for iLIRKO mice generation and euthanasia timepoints; (**B**) Timeline of the gene therapy approach with AAVs in iLIRKO mice and euthanasia timepoint.
